# Whole-Genome Sequencing for Routine Pathogen Surveillance in Public Health: a Population Snapshot of Invasive *Staphylococcus aureus* in Europe

**DOI:** 10.1128/mBio.00444-16

**Published:** 2016-05-05

**Authors:** David M. Aanensen, Edward J. Feil, Matthew T. G. Holden, Janina Dordel, Corin A. Yeats, Artemij Fedosejev, Richard Goater, Santiago Castillo-Ramírez, Jukka Corander, Caroline Colijn, Monika A. Chlebowicz, Leo Schouls, Max Heck, Gerlinde Pluister, Raymond Ruimy, Gunnar Kahlmeter, Jenny Åhman, Erika Matuschek, Alexander W. Friedrich, Julian Parkhill, Stephen D. Bentley, Brian G. Spratt, Hajo Grundmann

**Affiliations:** a Department of Infectious Disease Epidemiology, School of Public Health, Imperial College London, London, United Kingdom; b The Centre for Genomic Pathogen Surveillance, Wellcome Genome Campus, Hinxton, Cambridge, United Kingdom; c The Milner Centre for Evolution, Department of Biology and Biochemistry, University of Bath, Bath, United Kingdom; d School of Medicine, University of St. Andrews, St. Andrews, United Kingdom; e Pathogen Genomics, The Wellcome Trust Sanger Institute, Wellcome Trust Genome Campus, Hinxton, Cambridge, United Kingdom; f Department of Biology, Drexel University, Philadelphia, Pennsylvania, USA; g Programa de Genómica Evolutiva, Centro de Ciencias Genómicas, Universidad Nacional Autónoma de Mexico, Cuernavaca, Morelos, Mexico; h Helsinki Institute for Information Technology HIIT, Aalto, Finland; i Department of Mathematics, Imperial College London, London, United Kingdom; j Department of Medical Microbiology, University Medical Center Groningen, Rijksuniversteit Groningen, Groningen, The Netherlands; k National Institute for Public Health and the Environment (RIVM), Bilthoven, The Netherlands; l Centre Hospitalier Universitaire de Nice, Nice, France; m EUCAST Development Laboratory, Växjö, Sweden; n Department of Infection Prevention and Hospital Hygiene, Faculty of Medicine, University of Freiburg, Freiburg, Germany

## Abstract

The implementation of routine whole-genome sequencing (WGS) promises to transform our ability to monitor the emergence and spread of bacterial pathogens. Here we combined WGS data from 308 invasive *Staphylococcus aureus* isolates corresponding to a pan-European population snapshot, with epidemiological and resistance data. Geospatial visualization of the data is made possible by a generic software tool designed for public health purposes that is available at the project URL (http://www.microreact.org/project/EkUvg9uY?tt=rc). Our analysis demonstrates that high-risk clones can be identified on the basis of population level properties such as clonal relatedness, abundance, and spatial structuring and by inferring virulence and resistance properties on the basis of gene content. We also show that *in silico* predictions of antibiotic resistance profiles are at least as reliable as phenotypic testing. We argue that this work provides a comprehensive road map illustrating the three vital components for future molecular epidemiological surveillance: (i) large-scale structured surveys, (ii) WGS, and (iii) community-oriented database infrastructure and analysis tools.

## IMPORTANCE

The spread of antibiotic-resistant bacteria is a public health emergency of global concern, threatening medical intervention at every level of health care delivery. Several recent studies have demonstrated the promise of routine whole-genome sequencing (WGS) of bacterial pathogens for epidemiological surveillance, outbreak detection, and infection control. However, as this technology becomes more widely adopted, the key challenges of generating representative national and international data sets and the development of bioinformatic tools to manage and interpret the data become increasingly pertinent. This study provides a road map for the integration of WGS data into routine pathogen surveillance. We emphasize the importance of large-scale routine surveys to provide the population context for more targeted or localized investigation and the development of open-access bioinformatic tools to provide the means to combine and compare independently generated data with publicly available data sets.

## INTRODUCTION

Most bacterial infections of humans are caused by organisms that have a large population size and short generation times, and lineages with novel properties emerge and expand within observable time scales (1). Lineages that present a serious threat to public health are designated high-risk clones (HRCs) (2); these often combine enhanced virulence or transmission potential with multiple-antibiotic resistance. As HRCs are often difficult to treat and are associated with significant morbidity, mortality, and economic cost, they require targeted surveillance and containment at the population level. The considerable management challenge for public health microbiologists in tackling HRCs can be broken down into three tasks: (i) identification of public health risks posed by emerging and/or expanding HRCs, (ii) assessment of this risks by predicting important clinical and epidemiological consequences, and (iii) risk management through the implementation of prevention and control strategies. With the cost of sequencing entire bacterial genomes in steady decline and the development of powerful bioinformatic tools gathering pace, whole-genome sequencing (WGS) will inevitably be widely implemented for routine epidemiological surveillance. The dissemination of genomic data through established national and international networks of collaborating specialist laboratories will lead to increased awareness and shorter response times for HRCs. Ideally, these networks will be based on a shared bioinformatic infrastructure that links molecular data (genome sequences) and metadata (time, place, clinical details, and additional variables) with tools that help appraise the clinical and public health relevance of any given entry. This poses significant technical, ethical, and political challenges; however, the benefits of this fundamental shift in molecular epidemiology cannot be overstated. The efficient management and interrogation of genome data by public health and medical audiences may ultimately lead to the erosion of conventional reference diagnostic tasks such as identification to the species level, characterization of clinically important virulence or resistance phenotypes, and identification of outbreaks and inference of national or international transmission.

*Staphylococcus aureus* represents an epidemiological paradigm because of the undisputed public health relevance of this species, which is characterized by multiple-antibiotic resistance and a potential for swift dissemination through health care, social, and farm animal production networks. Here we demonstrate the utility of WGS when applied to a continental-scale representative “population snapshot” by using a novel data visualization platform. We sequenced 308 *S. aureus* isolates responsible for invasive infections that were recovered from 186 hospitals in 21 countries across Europe in a 6-month period. We consider three analytical strands: (i) a representative phylogeographic analysis that defines HRCs on a population level, (ii) an analysis of the dynamics of virulence and resistance carried by mobile genetic elements (MGEs), and (iii) an *in silico* ascertainment of antibiotic resistance encompassing 19 antibiotic compounds of clinical relevance.

To underline the added value for public health decision making when WGS data are supplemented with epidemiological metadata collected through structured surveys, we developed a web application (Microreact) that allows easy access and visualization of our data by medical and public health audiences and is available at the project URL (http://www.microreact.org/project/EkUvg9uY?tt=rc). This tool can be used for any appropriate data set where a phylogenetic tree and associated metadata are available (see http://www.microreact.org).

## RESULTS

We chose a random sample (*n* = 308; 10.6%) of isolates collected as part of a European structured survey of *S. aureus* from invasive diseases (3). Sixty percent (*n* = 185) of these isolates were methicillin-sensitive *S. aureus* (MSSA), and 40% (*n* = 123) were methicillin-resistant *S. aureus* (MRSA). A total of 235,226 SNP sites within the core genome were identified by mapping against a single reference genome, HO 5096 0412 (sequence type 22 [ST22]). We have divided the analysis and interpretation of the results into three parts: (i) a broad phylogenetic analysis and more fine-scaled consideration of exemplar lineages of high public health relevance, (ii) the distribution and dynamics of the accessory genes conferring virulence and resistance traits in the context of individual HRCs, and (iii) a comparison of antibiotic susceptibility profiles ascertained by *in silico* prediction from genome data with conventional susceptibility testing carried out in Staphylococcal Reference Laboratories (SRLs) and the European Committee on Antimicrobial Susceptibility Testing (EUCAST) Development Laboratory (EDL).

### Broad and fine-scale phylogenetic analyses for the identification of high-risk clones.

**FIG 1:**
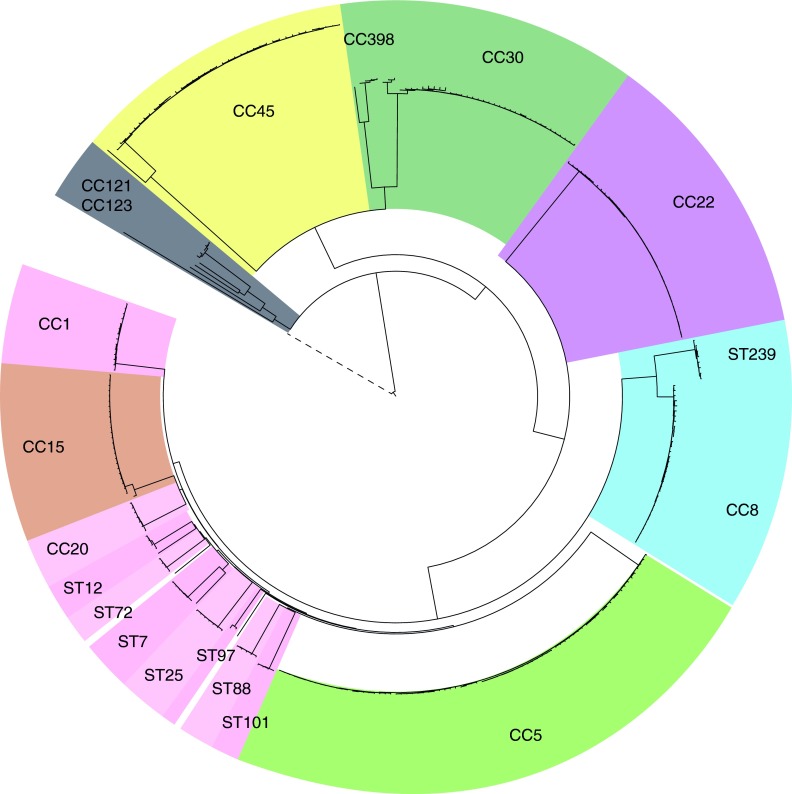
Phylogenetic relationship of the invasive *S. aureus* population circulating in Europe in 2006. A rooted neighbor-joining tree based on 235,226 genomewide core SNPs is shown. Lineages are highlighted and named according to the corresponding CC or ST.

**FIG 2:**
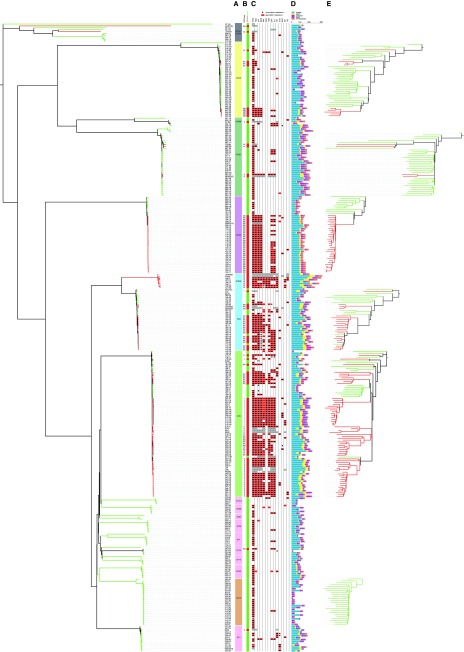
Phylogeny decoration. Colors of branches indicate MSSA (green) and MRSA (red) states. Each isolate is annotated by affiliation with a CC or ST (A), SSC*mec* type and MSSA (green) or MRSA (red) state (B), or antibiotic resistance profile (C). Red boxes indicate the presence of genetic resistance markers, black dots indicate phenotypic resistance, and gray boxes highlight resistance in reference genomes. (D) Size and composition of the accessory genome based on the number of noncore homologous groups with further categorization according to MGE type. (E) Close-up of phylogenetic trees of the six major lineages.

Analysis of core SNPs resolved the population into 6 major (CC5, CC22, CC8, CC30, CC45, CC15) and 10 minor clonal complexes (CCs) and STs (CC1, ST20, ST25, ST7, CC121, ST88, CC12, CC398, ST101, ST72), as previously defined by multilocus sequence typing (MLST)/eBURST (4) (Table 1; Fig. 1 and 2). The six major CCs are each represented by at least 24 isolates, whereas the minor lineages are represented by a minimum of four isolates and a maximum of 14. Subdivisions within a given CC and within individual STs are resolved by the data (Fig. 2E), which identify very closely related isolates resulting from recent expansion. We refer to these groups within CCs simply as clusters. These include many well-known MRSA “clones” previously defined by MLST, pulsed-field gel electrophoresis, and *spa* typing.

**TABLE 1:** Abundance, diversity, and proportion of MRSA isolates in each major or minor CC detected in the sample

Group and CC	Total no. of genomes^a^	No. of reference genomes	Proportion of MRSA genomes^b^	Mean no. of PW SNPs (SE)^c^	Mean yr of PW MRCA (range)^d^	Example clone(s)
Major						
CC5	78	8	0.8	438 (8.2)	1951 (1950–1952)	USA100 New York/Japan USA800, pediatric
CC22	41	1	0.775	266 (6.6)	1972 (1972–1973)	EMRSA-15, Barnim
CC45^e^	39	0	0.231	571 (9.4)	1935 (1933–1936)	USA600, Berlin
CC8^f^	33	5	0.642	456 (9.1)	1949 (1948–1950)	Iberian, USA300, USA500, archaic, Central European
CC30^g^	34	2	0.065	481 (5.8)	1946 (1945–1947)	EMRSA-16 (ST36), phage type 80/81, SWP, USA200
CC15^h^	24	0	0	258 (4.4)	1974 (1973–1974)	
Minor						
CC1	14	2	0	415 (9.3)	1954 (1953–1955)	USA400
ST20	7	0	0	369 (10.4)	1960 (1959–1961)	
ST25	7	0	0	307 (9.5)	1968 (1966–1969)	
ST7	6	0	0	159 (6.3)	1986 (1985–1987)	
CC121	5	0	0	737 (17.7)	1913 (1912–2016)	
CC88	5	0	0	356 (12)	1961 (1960–1963)	
CC12	4	0	0	365 (11.9)	1960 (1959–1962)	
CC398	4	1	0	326 (11.7)	1965 (1964–1967)	
ST101	4	0	0	240 (10.8)	1976 (1975–1978)	
ST72	4	0	0.25	275 (10.3)	1971 (1970–1973)	

a Including reference genomes.

b Excluding reference genomes.

c Mean number of SNPs in all possible pairwise (PW) combinations of genomes. Standard errors were estimated by bootstrapping (as implemented in MEGA v6.0). The standard error is the spread of pairwise values and reflects the degree of substructuring within each CC. A high standard error indicates that some pairs of genomes are closely related and others are more distant (that is, subclusters are apparent within the CC), whereas a low standard error indicates that all pairs of genomes show similar levels of divergence from each other and the phylogeny of the isolates within the complex is starlike. This analysis therefore indicates that, of the major CCs, CC15 exhibits the lowest degree of subclustering, whereas CC45 and CC8 exhibit the highest.

d Mean estimated date of the most recent common ancestor (MRCA) of all possible pairwise combinations of genomes. This is based on a mutation rate of 1.3 × 10^−6^ per site per year (or four SNPs per genome per year). This rate was proposed for ST22 by Holden et al. (8), and similar rates have been calculated for several other lineages (ST30, ST225, ST8-USA300) (6, 7, 9). We note that the mutation rate estimate for ST239 is approximately twice as high (3 × 10^−6^ per site per year) (12), for reasons that are unclear. The calculation is as follows. Approximating the genome size of *S. aureus* to 3 Mb, this rate equates to approximately four SNPs per genome per year. If a pair of genomes differs by, say, 500 SNPs, meaning 250 SNP changes, on average, in each of the two genomes, this would therefore correspond to 250/4 = 62.5 years of divergence. As the samples were collected in 2006, this means that the common ancestor of the two genomes would have existed around 1943.

e Excluding isolate 11_SE_395, as this is outside the main CC45 cluster.

f We have excluded the diverse ST239 genomes corresponding to the Portuguese, Brazilian, and EMRSA-4-7 clones, as this is a hybrid genome.

g Excluding ST34, as this is a hybrid genome.

h Excluding isolate 296_DE_582 (ST582), as this is a hybrid genome (see text).

The distribution of MRSA and MSSA isolates is not random with respect to CCs (Table 1). Ninety-three percent of the MRSA isolates belonged to only three CCs, CC22, CC5, and CC8, although these CCs represent only approximately half of the isolates (Table 1). Over 70% of the isolates in CC5, CC8, and CC22 are MRSA, whereas all of the isolates in CC15 are MSSA. CC45 shows moderate levels of MRSA (23%), and CC30 shows low levels (6%). The overall topology of the tree is highly consistent with previous studies (5) (Fig. 1 and 2). The impact of recombination on tree topology was investigated by computing the frequency of homoplasy (phylogenetic conflicts within the data). This confirmed previous work that revealed a high rate of homoplasy between the CCs (37.8% of the core SNPs) but very low levels when considering the variation within each of the CCs (0.62% of the core SNPs) (5). We are therefore confident that the fine-scaled phylogeographic inferences within individual CCs have not been seriously compromised by recombination. Intra-CC diversity was calculated simply as the mean number of SNP differences in all pairwise comparisons, and the average time since each pair of genomes shared a common ancestor was calculated on the basis of the published *S. aureus* mutation rate (Table 1) (6–9). This revealed similar levels of diversity between CCs and that, on average, the common ancestor of any pair of genomes belonging to the same major CC existed from the mid-1930s (CC45) to the mid-1970s (CC15).

A key observation from our previous work on *spa* typing was that MRSA variants tend to show geographic clustering, whereas MSSA variants do not (3). The WGS data permit more detailed comparisons of spatial signals within and between MRSA and MSSA clusters belonging to individual CCs. We examined three major CCs that illustrate the usefulness of WGS for public health and infection control; other CCs (CC45, CC8, ST239, and CC15) are discussed in the supplemental material (see Fig. S1 to S4, respectively). All of the phylogenetic analyses, presence/absence of accessory genes, and associated metadata are available at the project URL (http://www.microreact.org/project/EkUvg9uY?tt=rc) by use of the Microreact tool; a detailed guide is provided in Text S1 the supplemental material, with CC45 as an example.

### CC5.

**FIG 3:**
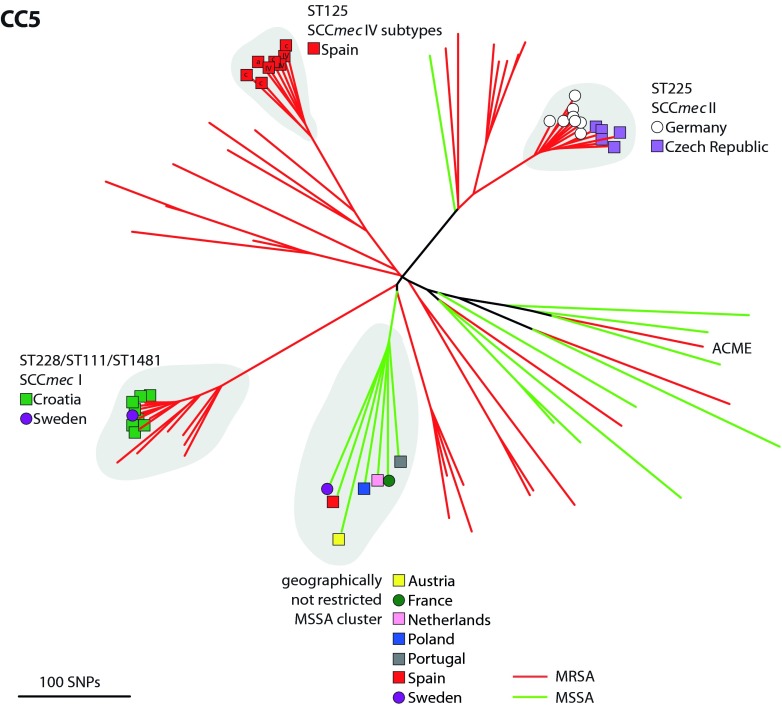
Phylogenetic reconstruction of CC5. Branch color indicates MSSA (green) or MRSA (red). Clusters described in Results are shaded gray. Symbols at the tips indicate the geographic origins of these isolates. SCC*mec* IV subtypes are shown for ST125.

CC5 is the most abundant CC in this study, being represented by 70 isolates, 80% of which are MRSA (red branches) (Fig. 3). Three MRSA clusters (ST225, ST228, and ST125) exhibit strong geographic structuring indicative of regional expansion. One MSSA (green branches) cluster is resolved on the tree but is not geographically restricted. This cluster largely corresponds to *spa* type t002, and this observation is consistent with our previous work showing that MSSA genotypes are widely distributed across Europe (3). The 13 isolates within the ST225 cluster all contain SCC*mec* type II elements. Seven of these originate from Germany, six are from the Czech Republic, and the partitioning within the phylogenetic tree coincides perfectly with the country of origin, although some of these isolates originate from areas close to the shared border (e.g., Ústí nad Labem in the Czech Republic and Grossenhain in Germany are just 122 km apart by road). The data thus point to the border between Germany and the Czech Republic as being a barrier to health care referral practices and thus MRSA spread.

The MRSA cluster corresponding to ST228 and related STs consists of 15 isolates all harboring a type I SCC*mec* element. Nine of these isolates define a very tight subcluster corresponding to ST111 (*n* = 8) and ST1481 (*n* = 1). Eight of these nine isolates originate from Croatia, which is indicative of rapid epidemic radiation in that country, and three isolates from Split cluster together on the tree, reflecting geographic structuring on a national level. One ST111 isolate originates from Sweden, consistent with the probable importation of ST111 into Sweden from Croatia by travel. The MRSA ST125 cluster consists of eight MRSA isolates restricted to central and northern Spain. For further investigation, see the Microreact tool, where supplementary analysis and geographic detail can be explored.

### CC22.

**FIG 4:**
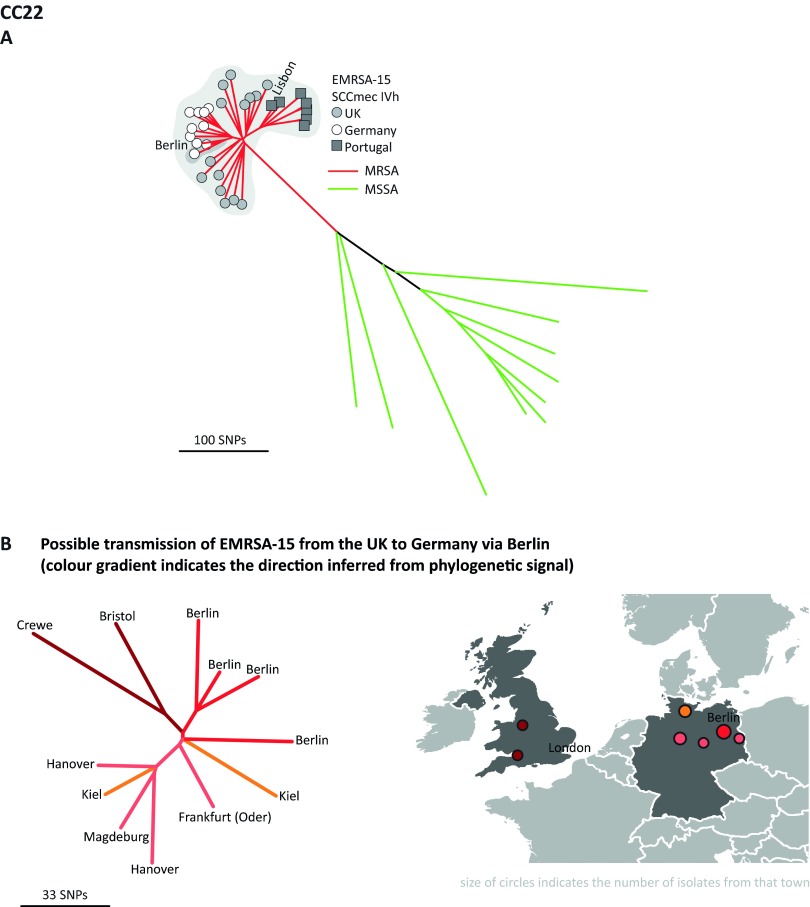
Phylogenetic reconstruction of CC22. Branch color indicates MSSA (green) or MRSA (red). The EMRSA-15 cluster is shaded gray. Symbols at the tips of the branches indicate the geographic origins of these isolates. A cluster consisting of isolates from Berlin indicating the possible point of EMRSA-15 introduction into Germany from the United Kingdom is shaded a darker gray. The position of an isolate from Lisbon is shown indicating the possible location of its entry into Portugal.

CC22 contains epidemic MRSA-15 (EMRSA-15) (Fig. 4A), which is currently the most abundant and fastest growing health care-associated MRSA (HA-MRSA) clone in Europe (10). This clone accounts for the majority of the CC22 isolates (31/41; 76%) in our sample, which are characterized by an SCC*mec* type IVh element and form a tightly clustered starlike phylogeny (Fig. 4A). Fourteen of these isolates originated from the United Kingdom, 11 were from Germany, and 7 were from Portugal. The topology of the tree supports a United Kingdom origin of EMRSA-15, followed by separate introductions from the United Kingdom into Germany and Portugal, consistent with the work of Holden et al. (8). In the German cluster, isolates from Berlin show the fewest SNP differences from the United Kingdom isolates, implicating that city as the initial point of introduction from the United Kingdom (Fig. 4B). This clone subsequently spread to the surrounding cities of Kiel, Frankfurt (Oder), Hanover, and Magdeburg, supporting epidemiological observations made by the Robert Koch Institute during investigations in the 1990s (11). Similarly, the Portuguese CC22 cluster points toward Lisbon as the point of introduction, with subsequent spread to Braga (in the far north) and Coimbra (central). All of the CC22 isolates located basal to the EMRSA-15 subtree are MSSA. They form a more diverse population, genetically and geographically, and represent the MSSA reservoir from which EMRSA-15 emerged (8).

### CC30.

**FIG 5:**
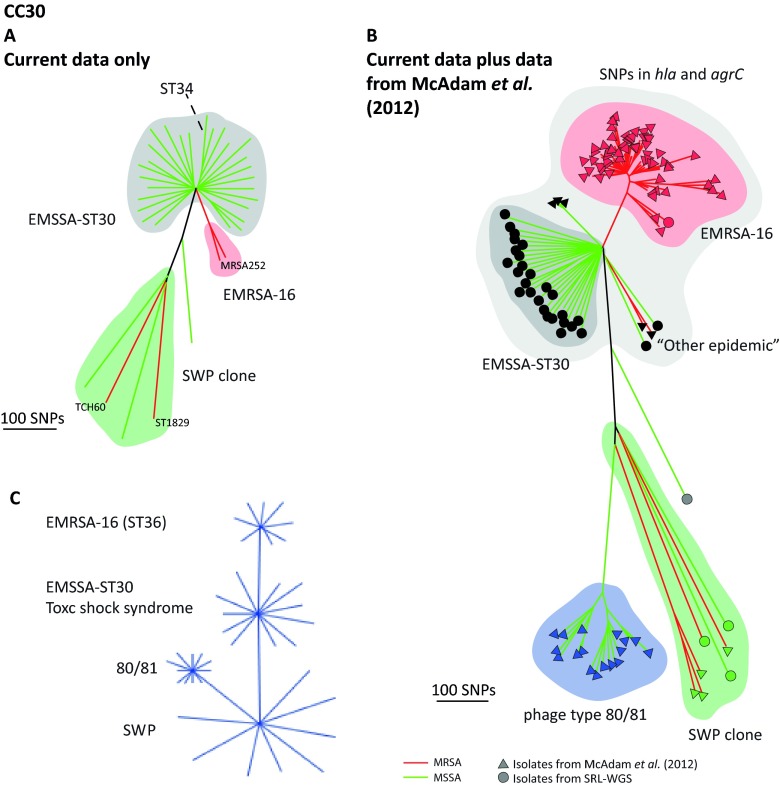
(A) Phylogenetic reconstruction of CC30 isolates in the sample. Branch color indicates MSSA (green) or MRSA (red). Reference genomes are named and clusters are highlighted and named in accordance with the report of McAdam et al. (7). The dashed line indicates the long branch leading to three ST34 isolates that evolved through the acquisition of a 200-kb homologous replacement within the chromosome from an ST10/ST145-like parent (66). (B) Phylogenetic tree of combined CC30 data obtained from isolates from the study of McAdam et al. (7) and isolates from panel A. Colors and cluster names are as in panel A. Light gray shading indicates isolates carrying SNPs in the *hla* and *agrC* genes thought to restrict these lineages to health care settings. (C) Representation of successive clonal radiations within the recent evolutionary history of CC30. These radiations correspond to recognized HRCs, both contemporary and historic. The SWP clone is a historic diversified starlike expansion with relatively long branches. Phage type 80/81 probably emerged from within this starlike expansion, as did the current MSSA HRC, which we have termed EMSSA-ST30. Finally, EMRSA-16 emerged from within the successful EMSSA-ST30 lineage, resulting in a more recent, and tightly clustered, starlike expansion.

CC30 contains successful HA-MRSA and community-associated MRSA (CA-MRSA) epidemic clones, including EMRSA-16 and the Panton-Valentine leukocidin (PVL) toxin-positive Southwest Pacific (SWP) clone. Figure 5A shows the tree for the 32 CC30 isolates present in the current sample and illustrates that only two (6.5%) were MRSA. One of the MRSA isolates is closely related to the MRSA252 reference, which is a HA-EMRSA-16 isolate (ST36), and the other (ST1829) is related to the TCH60 reference, a representative isolate of the SWP clone (14). The majority of 28 MSSA isolates form a striking starlike phylogeny with little geographic structure (for detailed investigation of geographic origin, see the Microreact tool). To place this lineage in a broader context, we combined our data with those of McAdam et al. (7), who recently sequenced representatives of the three major CC30 epidemic lineages; EMRSA-16, SWP, and the historical MSSA phage type 80/81 clone (Fig. 5B). This revealed that the large MSSA radiation corresponds to a successful progenitor population from which HA-EMRSA-16 emerged. This MSSA cluster, referred to by MacAdam et al. as “other epidemic,” is responsible for a significant disease burden in the community and in hospitals and encompasses a variety of *spa* types. Given the public health significance of this cluster, we tentatively suggest the designation EMSSA-ST30. All of the isolates in this cluster contain the SNPs in *hla* and *agrC* previously reported to attenuate virulence (18) (Fig. 5B). The presence of these SNPs among community-associated invasive disease isolates further challenges the suggestion that they play a role in nosocomial adaptation (18).

### Distribution and dynamics of accessory genes.

The accessory genome consists of genes that are variably present in a defined population and can be major drivers of adaptation (15). An understanding of the dynamics of key MGEs such as phages and the SCC*mec* elements, which carry determinants of the virulence and resistance phenotypes, is critical for monitoring the emergence and diversification of successful HRCs. We explored the diversity and distribution of accessory genes in our *de novo* assembled genomes by assigning genes to noncore homology groups (ncHGs). We categorized these groups according to the types of mobile elements with which they are associated, specifically, phages, SCC*mec* elements, plasmids, *S. aureus* pathogenicity islands, and transposons (Fig. 2). Fifty-seven percent of the ncHGs in the sample were phage associated, representing the most dominant category by far. The total number of ncHGs varies between different CCs; CC8 harbors the highest average number of ncHGs per isolate at 197, and CC15 has the lowest at 71 (see Fig. S5 in the supplemental material).

**FIG 6:**
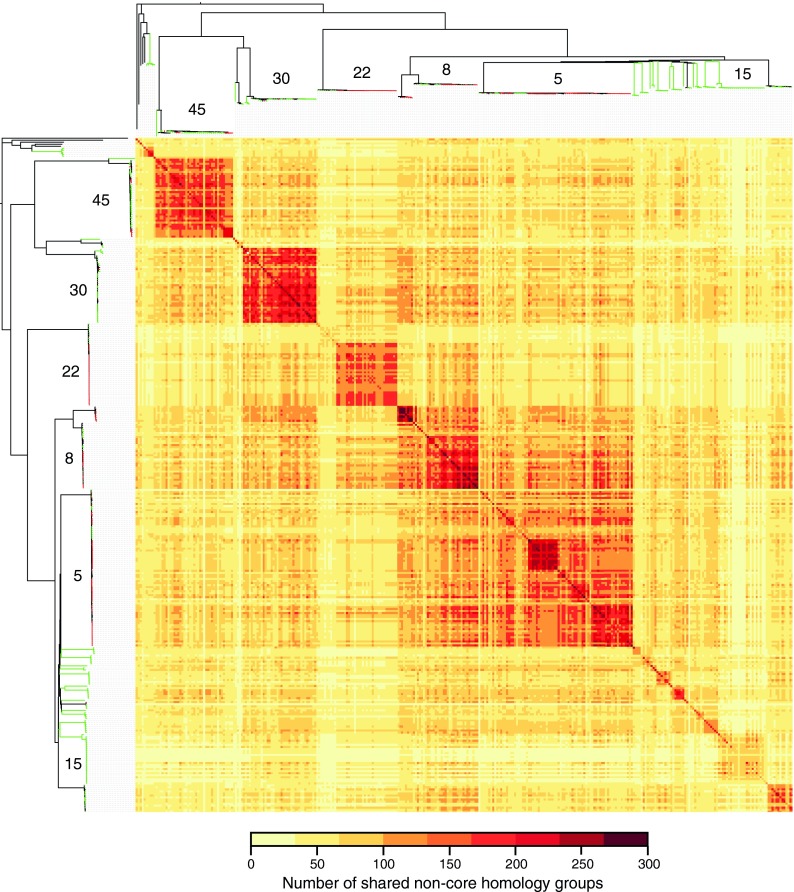
Conservation of ncHGs across the invasive *S. aureus* population in Europe. Isolates are arranged in the tree order of Fig. 2 along the left and top. The ncHGs in the pairwise comparison are displayed as a heat map matrix, and colors represent the total numbers of ncHGs that genome pairs have in common; darker shading indicates more ncHGs in common (see the scale at the bottom). The dark squares corresponding to (from the top) CC45, CC30, CC22, CC8, CC5, and (at the bottom) CC15.

Figure 2D illustrates the variation in the accessory genome within single CCs, and Fig. 6 shows the total number of ncHGs shared by each pair of genomes. Many more ncHGs are shared by isolates belonging to the same CC than by isolates belonging to different CCs. This supports the concept of a “core variable” genome (16), representing genes that are universally present at the scale of an individual CC but are variably present or absent with respect to the species as a whole. We also noted cases of MRSA clusters within CCs that have acquired a distinctive repertoire of ncHGs, evident as dark squares in Fig. 6; for example, the ST239 cluster (CC8), the ST228 cluster (CC5), and the ST225 cluster (also CC5). The high level of consistency between phylogenetic relatedness and the ncHG repertoire is expected over these very fine phylogenetic scales because of common inheritance. However, this consistency rapidly decays with increasing core genome divergence, particularly for highly dynamic elements such as phages. It is evident from Fig. 6 that CC5 and CC8 have a high number of ncHGs in common, resulting in a single large square that encompasses both CCs. The large proportion of MRSA strains within CC5 and CC8 does not, by itself explain, this observation, as it is still apparent when SCC*mec* is excluded (data not shown).

Comparing the total number of ncHGs within MRSA and MSSA isolates in the same CC revealed that MRSA clusters contain more ncHGs than closely related MSSA genomes, even when the SCC*mec* elements were excluded (see Fig. S6 in the supplemental material). For example, considering CC5 and CC22, MRSA isolates contain approximately 50% more ncHGs than MSSA isolates of the same complex. Although it is unclear whether the acquisition of SCC*mec* is the cause or the consequence of an increased propensity to integrate horizontally acquired DNA, this observation may be relevant to the finding that MRSA strains tend to contain many more resistance determinants than MSSA strains, conferring combined resistance to multiple classes of antibiotics.

Phages are important from a public health perspective, as they are carriers of virulence genes and resistance determinants. We assayed the distribution of seven phage types on the basis of the presence/absence of their integrase genes as described previously (17). The overall number of prophages per genome ranged from zero (9%) to five (0.3%), with a median of two (46%). Sa3*int* was the most commonly observed phage type, being present in 82% of our isolates, followed by Sa2*int* (32%), and Sa1*int* (27%). Sa4*int* was very rare, being observed in only four isolates (1.3%). The distribution of these phage types and associated cargo genes across the tree reveals important differences between the CCs and between different clusters belonging to the same CC (see Fig. S7 in the supplemental material). For example, the Sa3*int* prophage was found in all of the CCs, with the exception of CC15. This prophage can harbor four genes belonging to the immune evasion cluster, *chp*, *sak*, *scn*, and *sea*/*sep*, and in our data mostly harbored *sak* and *scn*, with the addition of either *chp* (e.g., CC22, CC45) or *sea*/*sep* (e.g., CC8, except USA300). Notably, *chp* and *scn* are common in CC15 even though the prophage itself is absent. The most likely explanation for this pattern is that the prophage was once integrated and then lost from the genome, leaving *chp* and *scn* behind.

SasX is a cell wall-anchored surface protein that is linked to the epidemiological success of ST239 in China and Southeast Asia (19). The ϕSPβ-like prophage that encodes SasX was identified among three ST239 isolates from Poland (see Fig. S7D). Two of these isolates cluster with the TW20 reference, which is representative of the “Asian clade” of ST239 (see Fig. S3), pointing to the introduction of these isolates into Poland from Asia. The third *sasX*-positive ST239 isolate in our data is more likely to be a native Polish variant and most likely acquired *sasX* from the imported Asian strains cocirculating in Poland. The data thus point to the horizontal dissemination, presumably via lysogenic conversion, of the phage-borne *sasX* virulence factor within Europe. Other examples of the horizontal dissemination of virulence genes are evident in our data. We note only a single representative of USA300, an MRSA strain from Belgium that closely clusters with the reference USA300 genome and contains a type I arginine catabolic MGE (ACME) (see Fig. S2). However, we also note a single CC5 SCC*mec* type V MRSA isolate from Portugal that has also acquired a type I ACME region (Fig. 3; see Fig. S7D). The distribution of other toxin and virulence genes, such as the presence of *tstH* in the cluster we designated EMSSA-ST30, is illustrated in Fig. S7D and incorporated into the visualization platform at the project URL.

Multiple SCC*mec* types (I, II, III, IV, V, and IV) were present among the 120 MRSA isolates. Type IV elements are the most widely distributed among different lineages, being present in all five of the major CCs that contain MRSA strains. From the phylogenetic analyses, we estimate that a minimum of 20 independent acquisitions of this element have occurred in our sample. However, the rate of SCC*mec* acquisition clearly varies between CCs, whereas only a single acquisition of the SCC*mec* type IVh element is observed in CC22, in CC5, there have been a minimum of seven different acquisition events encompassing five different SCC*mec* types. For example, the Spanish ST125 cluster harbors both SCC*mec* type IVc and IVs elements, indicating multiple acquisitions. In contrast, no CC15 isolates have acquired SCC*mec*. The reversion of MRSA to MSSA because of the deletion of SCC*mec* appears to be a relatively rare event and was identified in only two CC8 isolates in our sample (see Fig. S2).

### WGS as a tool for predicting antibiotic resistance.

The EDL tested all of the isolates against 16 antibiotics. In addition, all MRSA isolates were tested against a further three antibiotics that are prescribed mainly for the treatment of infections caused by MRSA. A total of 5,288 *in silico* predictions of resistance/susceptibility were made and compared against the EUCAST reference results in a blinded fashion. Of these, 1,075 were predicted to be resistant, compared with 1,050 identified phenotypically as resistant by the EDL, with concordance noted for 5,213 (98.6%) of the individual tests (Fig. 2; Table 2). However, there were some noteworthy exceptions. For the aminoglycoside amikacin, 21 isolates were falsely predicted to be resistant on the basis of the presence of the *aphA*-3 kanamycin resistance gene and *aadD*. It has previously been noted that the presence of these two aminoglycoside-modifying enzyme genes is a poor indicator of amikacin resistance (20, 21). Resistance to mupirocin was not predicted in five isolates that had inhibition zones (29 mm) close to the susceptibility breakpoint (≥30 mm). The reason for this discrepancy is likely to be normal variation or the choice of medium. There were also eight incorrect predictions of erythromycin susceptibility. In these cases, the result could readily be explained by loss of the *ermC*-carrying plasmid after sequencing but prior to phenotypic testing. Similarly, the loss of a BlaZ β-lactamase-carrying plasmid could explain the three false-negative predictions of penicillin resistance.

**TABLE 2:** Comparison of antibiotic resistances predicted by *in silico* and SRL test results against the EDL reference

Antibiotic	No. of *in silico* predictions vs EDL results					% Concordance	No. of SRL vs EDL results^a^		% Concordance	No. of *in silico* vs EDL results^a^		% Concordance
	Total	Traits	False positive	False negative	Discordant		Total	Discordant		Total	Discordant	
Penicillin	308	269	4	3	7	97.73	131	7	94.66	308	7	97.73
Cefoxitin	308	123	3	1	4	98.70	216	3	98.61	308	4	98.70
Ciprofloxacin	308	122	2	3	5	98.38	219	4	98.17	308	5	98.38
Moxifloxacin	308	118	2	0	2	99.35						
Amikacin	308	71	21	2	23	92.53						
Gentamicin	308	29	0	0	0	100.00	243	1	99.59	308	0	100.00
Tobramycin	308	77	7	0	7	97.73	79	1	98.73	308	7	97.73
Erythromycin	308	105	5	3	8	97.40	260	8	96.92	308	8	97.40
Clindamycin	308	95	3	2	5	98.38	172	10	94.19	308	5	98.38
Tetracycline	308	21	1	0	1	99.68	133	1	99.25	308	1	99.68
Tigecycline	308	0	0	3	3	99.03						
Fusidic acid	308	14	1	0	1	99.68	175	5	97.14	308	1	99.68
Linezolid	308	0	0	0	0	100.00	194	1	99.48	308	0	100.00
Mupirocin	308	9	0	5	5	98.38						
Rifampin	308	12	1	0	1	99.68	225	4	98.22	308	1	99.68
Trimethoprim	308	10	0	0	0	100.00						
Teicoplanin	120	0	0	3	3	97.50	87	3	96.55	120	3	97.50
Vancomycin	120	0	0	0	0	100.00	118	1	99.15	120	0	100.00
Daptomycin	120	0	0	0	0	100.00						
Total	5,288	1,075	50	25	75	98.58	2,252	49	97.82	3,628	42	98.84

a Only results for antibiotics tested by SRLs were compared.

We also determined the concordance of the antimicrobial susceptibility test (AST) results provided by the European *Staphylococcus* Reference Laboratories (SRLs), which contributed the isolates to our sample, with the EDL reference data. The SRLs submitted a total of 2,252 AST results. Of these, 2,203 (97.8%) were concordant with the EUCAST test results (Fig. 2; Table 2). Thus, it is clear that a small amount of discordance between phenotypic tests can be expected, even when they are carried out by experienced reference laboratories. We note that the degree of discordance between the *in silico* predictions and the EUCAST data is comparable to the degree of discordance between the two sets of phenotypic data. This demonstrates that *in silico* predictions were at least as reliable, in terms of matching the gold standard, as the AST results generated by independent reference laboratories.

## DISCUSSION

A consistent and comprehensive sampling frame is a crucial component of effective pathogen surveillance in public health, though in reality, this may involve significant logistical and political challenges. By taking a random sample of a larger collection assembled as part of a pan-European structured survey, we have minimized the problems associated with phylogenetic discovery bias (22) and are confident that our data capture a meaningful snapshot of *S. aureus* invasive disease isolates in Europe at the time of sampling. However, as with all sampling frameworks, important caveats remain. First, all of the isolates were recovered from invasive infections, primarily blood, meaning that we have underrepresented isolates causing skin and soft tissue infections. Second, laboratories submitted the first five MSSA isolates and the first five MRSA isolates from individual patients. A small number of laboratories did not receive five MRSA isolates during the 6-month survey because of a very low frequency of MRSA disease in their regions, for example in Scandinavia, and these laboratories completed their quota with MSSA isolates.

Despite these caveats, the combined analysis of WGS with epidemiological metadata addresses the following three key elements of managing infectious disease threats in public health.

### Genetic population structure and identification of HRCs.

The WGS data demonstrate that the disease-causing population of *S. aureus* is readily partitioned into highly discrete subpopulations or CCs. These CCs vary in their potential to spawn HRCs such as EMRSA-15 (8) or USA300 (9, 22), and our analysis suggests that these differences may be related to the varying propensity of each CC to acquire exogenous DNA (see Fig. S5 in the supplemental material). Analysis of fine-scale genetic variation and spatial structuring of the well-characterized HRCs circulating in Europe during the sampling period helped identify key population level properties, or HRC signatures, that can be used to recognize candidate HRCs, even in the absence of detailed phenotypic data (Fig. 3 to 5; see Fig. S1 to S4). Central to this is a consideration of phylogenetic tree topology, relative abundance, and geographic structuring.

Perhaps the most striking example is EMRSA-15, which emerged from the CC22 population approximately 30 years ago (8) and has subsequently become pandemic. Phylogenetic analysis of ST22 reveals a comet-shaped phylogeny, with two distinct parts; the comet head consists of the starlike radiation of EMRSA-15, reflecting recent rapid expansion, while the tail represents the more diverse MSSA progenitor population (Fig. 4A).

We find multiple similar signatures of rapid clonal expansions that coincide with recognized successful clones such as ST225, ST228, and ST125 within CC5 (Fig. 3). We also note previously unrecognized clusters in Europe that constitute candidate HRCs in the MSSA population, the most notable example being the CC30 MSSA designated EMSSA-ST30 here. By combining our data with the WGS data from a previous study of well-known CC30 HRCs (Fig. 5B), we placed EMSSA-ST30 within a broader evolutionary context. This revealed that the EMRSA-16 HRC emerged from EMSSA-ST30, as hypothesized originally from MLST data (24). This highlights the importance of recognizing successful MSSA lineages not only as HRCs in their own right but also in their role as likely progenitors of EMRSA (25).

### Assessing the risks posed by virulence and resistance determinants for public health.

CCs differ in their propensities to acquire and maintain accessory genome elements (see Fig. S1 in the supplemental material), a factor that may influence the emergence of HRCs. CC30 represents a good example of a lineage that has acquired elements carrying important virulence and resistance determinants. In addition to the phage-borne PVL toxin gene harbored in phage type 80/81 and the SWP clone, the cluster of isolates that we designated EMSSA-ST30 maintains the *tstH* gene encoding a superantigen that can cause toxic shock syndrome at high frequency (26) (see Fig. S7). This cluster is therefore reminiscent of, and likely descended from, the ST30 variants responsible for the tampon-associated toxic shock syndrome that emerged in the 1980s (27). This provides another line of evidence for the public health risk posed by this widespread clone.

The success of ST239 in China and much of Southeast Asia has been largely attributed to SasX, a surface-anchored protein that modulates host interactions and transmissibility and is carried on the ϕSPβ-like prophage (19). In Poland, we found evidence that ST239 isolates from the successful Asian lineage spread repeatedly to Europe (12) but also that this *sasX* virulence determinant has been transduced from the European ST239 genetic background (see Fig. S3). This observation justifies heightened epidemiological vigilance with respect to this HRC, not only for particular epidemiologically successful clones but also for virulence determinants they can spread.

A key characteristic of HRCs has been the emergence of antibiotic resistance. WGS provides the opportunity to scan genomes for all known genetic determinants of antibiotic resistance. We examined the distribution of known resistance determinants and the extent to which it is possible to predict resistance profiles from sequence data in a blinded fashion. Whereas previous studies have shown a high concordance between phenotypic data and genotypic predictions (8, 13, 28), we sought to extend these studies by including a greater number of antibiotics (19 in total) and by including a representative sample of isolates that cause invasive disease among patients in European hospitals and that we consider the clinically most relevant representatives of the *S. aureus* population. Further, as discrepancies between the two methods could arise from uncertainties in the phenotypic data, as well as inaccurate predictions from genotyping, we compared two independent sets of phenotypic data, one contributed by various SRLs and the second generated at the EDL. Taking the EDL data to represent the “gold standard,” our genotypic predictions show higher rates of concordance with these data (EDL versus genotype, 98.6%) than do the data generated by the SRLs (EDL versus SRL, 97.8%, Table 2). We conclude that genotypic prediction is at least as reliable as routine phenotypic testing and that any discrepancies between the two approaches are just as likely to represent inaccuracies in the phenotypic testing as inaccurate genotypic predictions.

We do not, however, propose that genotypic prediction should replace phenotypic testing, as such a strategy would be vulnerable to the emergence of new and uncharacterized antibiotic resistance mechanisms. Moreover, *in silico* prediction for other organisms has proved more challenging, especially for Gram-negative bacteria (29), where the present understanding of the genetic basis of resistance is less comprehensive. Despite these limitations, WGS data clearly provide additional objective evidence that is not prone to heterogeneities inherent to conventional phenotypic test methods deployed in different reference laboratories. Moreover, in the longer term, WGS will deliver a digitized and cumulative record that will address the need for internationally agreed standards for collection of data and reporting on antibacterial resistance in human health and for harmonizing standards across medical, veterinary, and agricultural sectors as required by the WHO, the World Organization for Animal Health, and the Food and Agriculture Organization of the United Nations (30).

### Deployment of informed and targeted prevention and control strategies.

We have argued for a two-pronged approach to the identification of emerging HRCs and an assessment of the threats they pose to public health, (i) a consideration of clonal relatedness, abundance, and phylogeographic structure at the population level and (ii) mining of the accessory genome repertoire to ascertain likely virulence and resistance properties. These two perspectives should also be combined when considering appropriate containment measures. The recognition of an HRC through large-scale surveillance would enable the development of tailored rapid diagnostic tests based on distinguishing SNPs or accessory gene signatures. This will allow for selective screening and targeted containment by decontamination and isolation strategies that are easier to implement, more economical, and more likely to be effective than more generic procedures. The expedition of specifically tailored tests during outbreaks would allow health authorities or infection control practitioners to screen potential hosts for colonization or infection, thereby reducing the chance of onward transmission. WGS allows for rapid appraisal of outbreak sources and transmission pathways that will also help in weighing up infection control priorities. Moreover, information regarding the virulence gene repertoire associated with an emerging or outbreak HRC will inform clinicians and medical microbiologists of likely clinical manifestations.

Ultimately, the key to managing HRCs lies in making the data available in an open and intuitive format for infection control and public health audiences. This democratization of the data increases the collective power of interpretation (i.e., the identification of HRCs) while decreasing the necessity for local expertise in bioinformatics. The coupling of large-scale population sampling by WGS within open-access and freely available web resources empowers the community to identify clone-specific signatures (canonical SNPs and/or accessory genes), promoting the design of HRC-specific rapid and cost-effective molecular diagnostic tests (31).

With this communication, we aimed to demonstrate how the integration of WGS into epidemiological surveillance programs provides the means for both the early warning of emerging HRCs and a robust assessment of associated public health threats. However, the advent of the underlying sequencing technology addresses only a small part of the challenge of managing the threat from infectious disease. In order to exploit this technical advance to its maximum potential, two things must happen. First, national reference laboratories need to agree to, and abide by, common standards for the contribution of isolates to structured surveys, ideally at 5-year or shorter intervals, on pancontinental and, ideally, global scales. Second, platforms for bioinformatics need to be developed to allow intuitive management and exploration of the data. These data sets should conform to internationally curated standards for sets of genes and mutations that are recognized as key virulence or resistance determinants, and we propose the initiation of internationally curated data sets to act as a gold standard resource for genomic antimicrobial resistance determinants.

The establishment of cumulative databases will engender a far richer understanding of the detailed dynamics underpinning clonal emergence and replacement, national and international transmission, and the horizontal transfer of core genes and MGEs. The increasing public health threat from bacterial pathogens is, in large part, down to the ability of these organisms to rapidly adapt through the dissemination of genes and mobile elements. Our best chance of managing these threats in the future is to emulate, as far as possible, this resource and data sharing through the development of international surveillance networks and a common data exchange infrastructure.

## MATERIALS AND METHODS

### Sampling framework and bacterial isolates.

From September 2006 to February 2007, 357 laboratories serving 450 hospitals in 26 countries collected nearly 3,000 MSSA and MRSA isolates from patients with invasive *S. aureus* infections, as described previously (3). Approximately 10% (*n* = 308) of these were randomly selected for sequencing. Isolate details are available for download at the project URL.

### Genomic library creation and sequencing.

For each sample, index-tagged libraries were prepared and sequenced in Illumina Genome Analyzer GAII cells with 54-base paired-end reads or Illumina HiSeq with 75-bp paired-end reads. Downstream processing utilized the index tag sequence information to assign reads to individual samples.

### Reference genomes.

To provide a wider context to the data, we utilized 26 fully annotated complete *S. aureus* reference genomes. The strain names and accession numbers are as follows: 04-02981, CP001844 (6); COL, CP000046 (32); ECT-R2, FR714927 (33); ED133, CP001996 (34); ED98, CP001781 (35); HO 5096 0412, HE681097 (8); JH1, CP000736 (36); JH9, CP000703 (36); JDK6008, CP002120 (37); JKD6159, CP002114 (38); LGA251, FR821779 (39); MRSA252, BX571856; MSSA476, BX571857 (40); Mu3, AP009324 (41); Mu50, BA000017 (42); MW2, BA000033 (43); N315, BA000018 (42); NCTC8325, CP000253 (44); Newman, AP009351 (45); RF122, AJ938182 (46); BB155, LN854556; ST398, AM990992 (47); TCH60, CP002110; TW20, FN433596 (48); USA300_FPR3757, CP000255 (23); USA300_TCH1516, CP000730 (49).

### Detection of SNPs in the core genome.

The paired-end reads for the survey isolates and the 26 publicly available genomes were mapped with SMALT (http://www.sanger.ac.uk/resources/software/smalt/) against the chromosome of *S. aureus* strain HO 5096 0412 (EMRSA-15, ST22; accession number HE681097) (8). The reference genomes were mapped by using simulated paired fastq data (54-bp paired ends with a 300-bp insert). SNPs were identified as previously described (50). Indels were identified with the Genome Analysis Toolkit (https://www.broadinstitute.org/gatk/) (51). Unmapped reads and sequences that were not present in all of the genomes were not considered part of the core genome, and SNPs from these regions were not included in the phylogenetic analysis. SNPs falling within MGE regions were also excluded from the phylogenetic analysis, as described previously (8).

### Phylogenetic analysis.

Phylogenetic analysis of the isolates combined was carried out by the neighbor-joining method as implemented in FastTree (52). In cases where data were combined with already published data, RAxML v8.0 (53) was used for tree reconstruction based on maximum likelihood. Only SNPs corresponding to the core genomes were used for phylogenetic analysis. *In silico* reconstruction of STs conventionally generated by MLST for each isolate was carried out with the sequence data as described by Croucher et al. (50). To root the tree, we used the sequence of BB155, an *S. aureus* reference genome that belongs to ST152. This was considered an appropriate outgroup as, according to MLST data, ST152 is a divergent lineage approximately equidistant from each of the major *S. aureus* STs and CCs previously recorded in Europe (54).

### Pangenome analysis.

Assemblies were produced with a pipeline comprising three steps: (i) correction of sequencing errors in reads with Quake v0.3 (55), (ii) *de novo* assembly of corrected reads into scaffolds with SOAPdenovo2 (56) by using a kmer of 31, and (iii) remapping of the corrected reads to fill scaffold gaps with GapCloser v1.12 (56). Prediction of protein coding sequences (CDSs) in assemblies was done with Prodigal (57), and FASTA sequences and coordinate positions were extracted. An all-versus-all BlastP (58) search of translated sequences was used as the input for the definition of homology groups (HGs) with TribeMCL (59) (cutoff, 1e-50) with group membership stored for querying. From a total of 749,089 putative CDSs, 4,281 HGs were defined with various numbers of members (1 to 1,827). Core HGs were defined as being present in at least a single copy in each assembly, and all others were considered ncHGs. SCC*mec* elements were typed by mapping reads to a pseudomolecule of the known SCC*mec* variants and also by comparative analysis using the assemblies.

### Detection of prophages and virulence genes.

Prophages were classified by using conserved areas of the integrase (*int*) genes as described previously (17) by an *in silico* PCR approach. Virulence and toxin genes of public health interest were similarly detected through *in silico* searches for previously published primer sequences (*eta* [60]; *luk-PV* [61]; *chp*, *sak*, *scn*, *sea*, *sep*, *hlb* [62]; *lukM* [63]; *sasX* [18]; *tstH* [64]; *acr* [65]).

### Genomic prediction of antibiotic resistance.

Resistance profiles for 19 antibiotics were predicted *in silico* from the sequence data as previously described (8). In brief, the literature was comprehensively mined for the known genetic mechanisms of antibiotic resistance in *S. aureus* (see Table S1 in the supplemental material). Antibiotic resistance conferred by SNPs in components of the core chromosome were identified from the mapping data. Antibiotic resistance conferred by accessory genes was identified by comparing *de novo* assemblies against a database of *S. aureus* resistance genes with BlastN (58) and by mapping sequence reads to a pseudomolecule consisting of concatenated antibiotic resistance genes as detailed by Holden et al. (8).

### Phenotypic testing of antibiotic resistance.

The antibiotic susceptibility of all *S. aureus* isolates was tested by the standardized EUCAST disk diffusion method in the EDL, Växjö, Sweden, without prior knowledge of the sequence data or metadata. Full methodological details are available in the EUCAST Disk Diffusion Test Manual, v 3.0, 2013 (http://www.eucast.org). Phenotypic resistance was defined by applying inhibition zone diameter epidemiological cutoff values (ECOFFs) and EUCAST clinical breakpoints (EUCAST breakpoint tables for interpretation of MICs and zone diameters, version 3.1, 2013 [http://www.eucast.org]). We pooled the intermediate and resistant categories into a single nonsusceptible category. For our sample, this partition was fully consistent with the classification into wild-type and non-wild-type isolates defined by the EUCAST ECOFFs. The antibiotics tested were penicillin, cefoxitin, ciprofloxacin, moxifloxacin, amikacin, gentamicin, tobramycin, erythromycin, clindamycin, tetracycline, tigecycline, fusidic acid, linezolid, mupirocin, rifampin, and trimethoprim. MRSA resistance to clinically relevant reserve antibiotics (teicoplanin, vancomycin, and daptomycin) not suitable for disk diffusion testing was determined by using the respective MIC ECOFFs (2.0, 2.0, and 1.0 mg/liter, respectively) with E tests (bioMérieux Clinical Diagnostics, Marcy l’Etoile, France). Phenotypic test results were dichotomized and grouped into wild-type and non-wild-type categories. Results of genomic predictions were compared with the phenotypic classifications as resistant or susceptible as defined by the ECOFFS. Moreover, results that were made available by the SRLs were equivalently dichotomized and also compared to those generated by the EDL in a consistent manner.

### Web application.

Microreact is a Node.js application written in JavaScript. Locational data are displayed by using the Google Maps Application Programming Interface, and phylogenetic data (in Newick tree format) are displayed by using a custom tree viewer developed for the HTML5 Canvas element (PhyloCanvas). Image files (.png) of annotated trees and subtrees can be saved. Instructions and examples are shown at the microreact.org website. The project URL for exploring the data described in this article is http://www.microreact.org/project/EkUvg9uY?tt=rc.

### Nucleotide accession numbers.

WGS data for all of the isolates tested in this study have been deposited in the Sequence Read Archive, and accession numbers are included in the metadata available at the project URL.

## SUPPLEMENTAL MATERIAL

Text S1  Appendix: Walkthrough of web application for genomic pathogen surveillance. Download Text S1, DOCX file, 1.2 MB

Figure S1  Phylogenetic reconstruction of CC45. Branch color indicates MSSA (green) or MRSA (red). The symbols at branch tips indicate the geographic origins of these isolates. SCC*mec* IV subtypes are shown. Download Figure S1, PDF file, 0.2 MB

Figure S2  Phylogenetic reconstruction of CC8 (excluding ST239). Branch color indicates MSSA (green) or MRSA (red). The symbols at branch tips indicate the geographic origins of these isolates. Close-ups of the two SCC*mec* IV clusters show their starlike radiation. SCC*mec* IV subtypes are shown. The third cluster represents USA300 isolates consisting of two reference isolates and one Belgian isolate. Download Figure S2, PDF file, 0.2 MB

Figure S3  (A) Phylogenetic reconstruction of ST239 isolates in the sample. Red branches depict all of the MRSA isolates. The symbols at the tips of the branches indicate the geographic origins of the isolates. Isolates carrying the *sasX* gene are indicated by the letter X. Blue shading highlights isolates belonging to the Asian clade according to Harris et al. (S. R. Harris, et al., Science 327:469-474, 2010). (B) Phylogenetic tree of combined ST239 data obtained from isolates from the reports of Harris et al. and Castillo-Ramírez et al. (S. Castillo-Ramírez, et al., Genome Biol 13:R126, 2012) and isolates in panel A. Clusters are named in accordance with the reports of Harris et al. and Castillo-Ramírez et al. Download Figure S3, PDF file, 0.2 MB

Figure S4  Phylogenetic reconstruction of CC15. Starlike phylogeny indicates the recent rapid expansion of this MSSA-only lineage. Symbols at the tips indicate the geographic origins of these isolates. The branch leading to an ST582 isolate—a single-locus variant of CC15—is shortened, and the number of SNPs defining that branch is shown. Download Figure S4, PDF file, 0.2 MB

Figure S5  Number of ncHGs per major lineage. The bar chart shows the average number of ncHGs per isolate split into MGE type for each of the six major lineages. Each pie chart indicates the proportion of MGE types for each major lineage. Download Figure S5, PDF file, 0.2 MB

Figure S6  MRSA or MSSA genome size. The bar chart shows the average number of ncHGs per MSSA or MRSA isolate, respectively, excluding the HGs associated with SCC*mec*, for each major lineage. Asterisks indicate lineages with a significant size difference of the accessory genome in MSSA and MRSA isolates. CC8, *P* = 0.039; CC5, *P* = 0.0325 (statistically significant); CC22, *P* = 0.0539 (not quite statistically significant). Download Figure S6, PDF file, 0.03 MB

Figure S7  Prophage distribution. Rooted neighbor-joining tree like that in Fig. 1. Colors of branches indicate MSSA (green) and MRSA (red). Each isolate is annotated for affiliation to CCs or STs (A), SSC*mec* type and MSSA (green) or MRSA (red) (B), and seven prophage types classified on the basis of the presence or absence of their integrase genes (C). Colored boxes indicate presence. Black boxes indicate the presence of virulence genes associated with the prophage. (D) Colored boxes indicate the presence of two virulence genes (*sasX* and *tstH*) and *acr*—the gene commonly used to detect ACMEs. (E) Size and composition of the accessory genome based on the number of ncHGs with further categorization according to MGE type. (F) Close-up of phylogenetic trees of the six major lineages. Download Figure S7, PDF file, 0.8 MB

Table S1  Known genetic mechanisms of antibiotic resistance in *S. aureus* used to predict genotypic resistance. Table S1, XLSX file, 0.04 MB

Table S2  Affiliations of and contact information for members of the European SRL Working Group. Table S2, XLSX file, 0.02 MB
