# Supplementary material for: Whole-Genome Sequencing for Routine Pathogen Surveillance in Public Health: a Population Snapshot of Invasive Staphylococcus aureus in Europe
Source: mBio. 2016 May 5;7(3):e00444-16. doi: 10.1128/mBio.00444-16 (PMC4959656; doi:10.1128/mBio.00444-16)
Supplement: Text S1 — Appendix: Walkthrough of web application for genomic pathogen surveillance. Download [file mbo002162813s1.docx]

**APPENDIX : Walkthrough of Web Application for Genomic
 Pathogen Surveillance**

Data generated as part of the present study are accessible through a web application located at http://www.microreact.org/project/EkUvg9uY?tt=rc. The application is compatible with any up to date web browser (eg IE 9+, Firefox, Safari), though for best results we recommend using Google Chrome (http://www.google.com/chrome/).

**Using the web application**:

The application contains three main sections: a map (A), a tree viewer (B) and a menu allowing detailed interrogation of the metadata (C) All three sections can be expanded by dragging the boundaries to display a larger view. To navigate within the map and tree sections simply use the mouse scroll wheel (or equivalent) or the standard zoom and pan controls in the map window.

In the start-up view, the locations of all laboratories that contributed to the sample are displayed on the map (A). The inferred phylogeny of all sampled isolates is displayed within the tree viewer to the right of the map (B). By default, points on the map, and nodes on the tree, are coloured based on country of origin. Clicking on the ‘table’ icon within the menu (C) will reveal the data for all the isolates currently shown on the map (all isolates by default, scroll to view). Selecting a point on the map will open an ‘Info Window’ indicating the isolate IDs from that location, and will filter the data in the ‘Metadata Table’ to show the information only for those isolates contributed by the selected laboratory. Interaction between the map and the tree works in two directions; Firstly, clicking a node (or internal branch) on the tree will show locations on the map which correspond to the origins of isolates on the selected branch, and simultaneously the isolate metadata. Secondly, clicking a point on the map and then an Isolate ID will highlight (in blue) the terminal node on the tree representing that isolate (best seen when tree zoomed). The highlighted tree node(s) and branches can be deselected simply by clicking outside of the tree, which will return the full set of points to the map. All the points within a defined region of the map can be selected using the map region filter tool in the top right of the map panel.

The field for detailed interrogation (in the lower part of the screen; C) can be accessed by clicking the ‘eye’ menu icon. This view is split into the left hand side (‘’Shapes and Colours’) and the right hand side (‘Labels’). The left hand side provides two options for colouring of the points on the map and on the nodes on the tree; either according to country (the default setting) or with respect to MRSA status. If the MRSA option is selected, RED points (on the map), or nodes (on the tree) represent MRSA and green represents MSSA. On the map, yellow points represent laboratories that have sampled both MRSA and MSSA.

The “Labels” section on the right hand side (D) provides different options for changing the text (labels) on the tree according to different metadata fields, but this does not change the map. For example, ‘MLST’ from the Label section places designated MLST sequence types (STs) as text next to each tree node. The same can be done for the *spa* type, or SCC*mec* type etc. Genetic resistance determinants (genes or mutations leading to resistant phenotypes), or the presence of toxins and phage types can also be viewed on the tree. Tree labels can be toggled on or off by clicking the right mouse button anywhere within the tree panel to bring up a sub-menu.

**Navigating the tree**:

The tree type (Rectangular, Circular or Radial) can be changed using the ‘Tree Type’ icons (E). Node size and label size can be adjusted using the sliders. Zooming down to visualize fine details of the branching patterns and overlaying metadata can be achieved simply by using the mouse scroll wheel (or equivalent). It is also possible to display a sub-tree separately. First, place the cursor over the internal node defining the sub-tree of interest. Once a blue circle appears, left-click on the node to highlight the subtree in blue. Once a sub-cluster is highlighted, right-clicking on the subtree will bring up a sub-menu with the following options containing options to manipulate the tree. Selecting ‘Redraw subtree’ from the sub-menu will replace the current view with the selected sub-tree view, and will simultaneously filter the points on the map so that only those corresponding to the isolates sub-tree are given. In order to step back to a previous tree, click on the “>” symbol located towards the top of the partition between the tree window and the map window. This opens a “tree-history” panel which allows you to select any of the recently viewed subtrees. Selecting “Redraw Original tree” at any point will take you back to the default tree view, though this will not wipe the tree history. Screenshots can be generated via the download icon (top right) as well as downloading all data (nwk tree file and csv metadata file).

**Walkthrough of CC45**:

The CC45 complex is the first major complex located at the top of the tree (in the same position as in Figure 2). To locate this cluster, zoom in to the top part of the tree (using the central scroll wheel), click ‘MLST’ within the ‘colours and labels’ tab (E) and increase the label size using the horseshoe slider. To view the CC45 complex in more detail, right click on the internal node at the base of the CC45 (excluding the more distant ST395) and select ‘Redraw Subtree’. This will change the tree view to the subtree for CC45, and the points on the map are now filtered to show only those points corresponding to laboratories that collected CC45 isolates. Change the tree type to radial for ease of viewing. Clicking the ‘country’ label will display country of origin on the tree, and click the MRSA button in the ‘Shapes and Colours’ section to highlight the nodes as either red (MRSA) or green (MSSA). The MRSA and MSSA clusters described in the text should now be visible. Other metadata labels can be overlaid on the tree, such as *spa* or SCC*mec* type. Finer geographic sub clustering (as described in the text) can be viewed by selecting the ‘City’ Label and/or zooming in on the map.
