# Supplementary material for: Whole-Genome Sequencing for Routine Pathogen Surveillance in Public Health: a Population Snapshot of Invasive Staphylococcus aureus in Europe
Source: mBio. 2016 May 5;7(3):e00444-16. doi: 10.1128/mBio.00444-16 (PMC4959656; doi:10.1128/mBio.00444-16)
Supplement: Figure S1 — Phylogenetic reconstruction of CC45. Branch color indicates MSSA (green) or MRSA (red). The symbols at branch tips indicate the geographic origins of these isolates. SCCmec IV subtypes are shown. Download [file mbo002162813sf1.pdf]

CC45

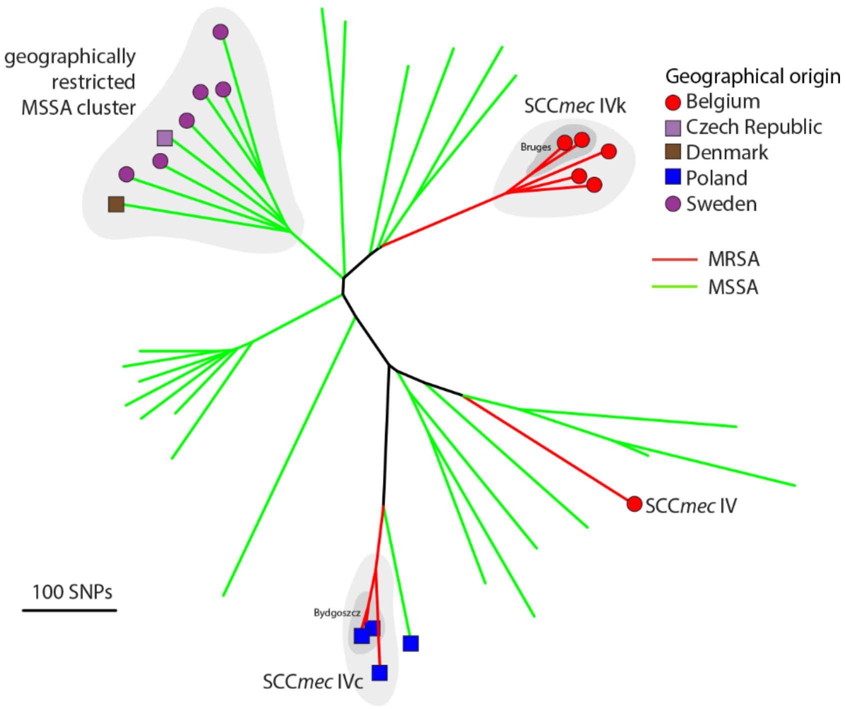

Although CC45 is predominantly MSSA, our data indicate three independent acquisitions of SCC*mec*, two of which have been followed by regional spread, evident as clusters on the tree. Five related isolates from Belgium have acquired an SCC*mec* type IVk element, and three related isolates from Poland have acquired an SCC*mec* type IVc element. A single unrelated isolate from Belgium has acquired an SCC*mec* type IV element. Closer inspection of the two MRSA clusters reveals evidence for geographic structure at national scale. Two Belgian isolates from a single hospital in Bruges are more closely related to each other than the other Belgian isolates. The same applies to two isolates from Bydgoszcz, Poland (also from the same hospital), which are more related to each other than to other Polish isolates. A geographically restricted cluster consisting entirely of eight MSSA isolates is also apparent in CC45, six of which are from Sweden, one from Denmark and one from the Czech Republic. While our previous study [3], based on *spa* typing, provided little evidence of geographic structuring among MSSA isolates, this observation illustrates the superior power of WGS data for detecting recent emergence and localised spread, even within MSSA clusters.
