# Supplementary material for: Whole-Genome Sequencing for Routine Pathogen Surveillance in Public Health: a Population Snapshot of Invasive Staphylococcus aureus in Europe
Source: mBio. 2016 May 5;7(3):e00444-16. doi: 10.1128/mBio.00444-16 (PMC4959656; doi:10.1128/mBio.00444-16)
Supplement: Figure S2 — Phylogenetic reconstruction of CC8 (excluding ST239). Branch color indicates MSSA (green) or MRSA (red). The symbols at branch tips indicate the geographic origins of these isolates. Close-ups of the two SCCmec IV clusters show their starlike radiation. SCCmec IV subtypes are shown. The third cluster represents USA300 isolates consisting of two reference isolates and one Belgian isolate. Download [file mbo002162813sf2.pdf]

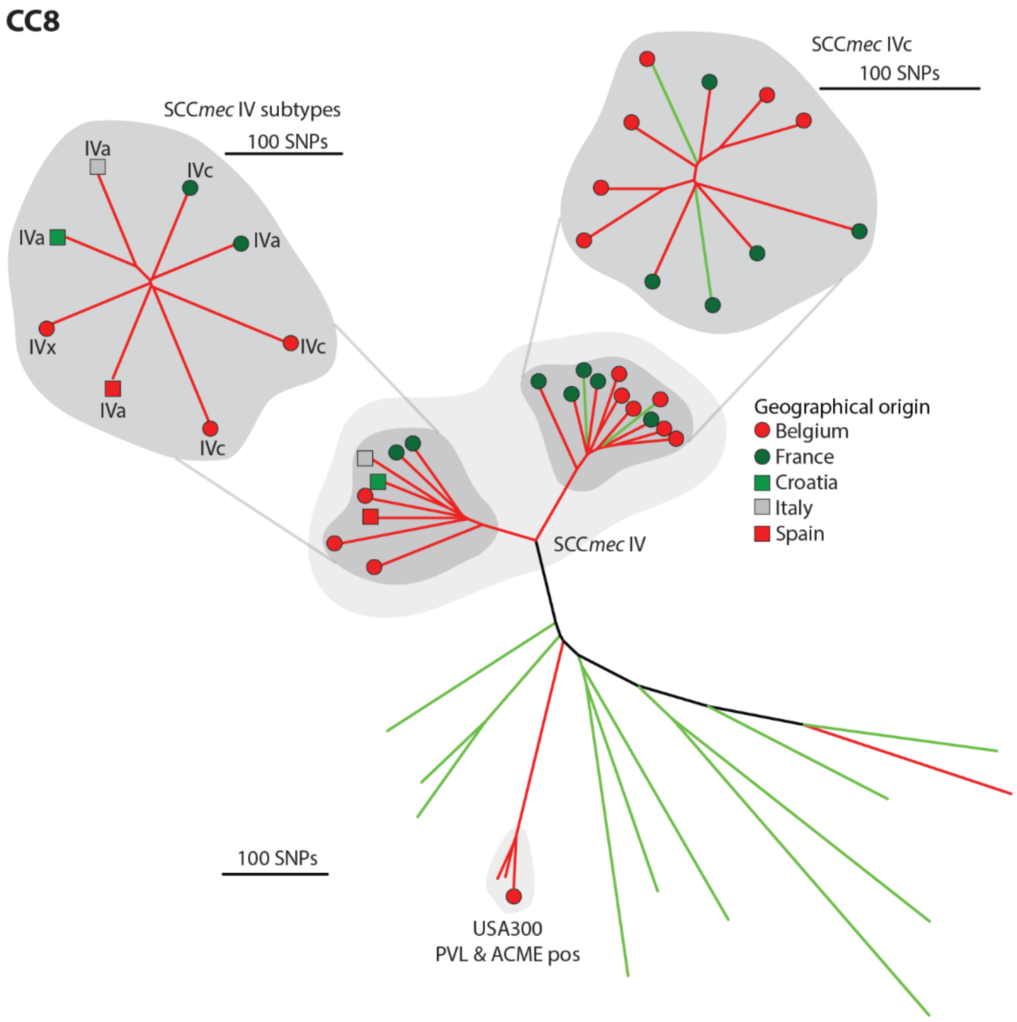

CC8 encompasses a number of international community-acquired as well as HA-MRSA lineages, including USA-300 which is a widespread CA-MRSA clone (and increasingly an HA-MRSA clone – e.g. in North America [53]), and ST239 which is a globally distributed HA- MRSA clone [54]. ST239 emerged via a large (625 kb) homologous replacement from a CC30-like donor [22,55], which accounts for the significant distance on the tree between ST239 and the rest of CC8 (Figure 1). Given the global importance of ST239, we shall first consider the other CC8 isolates first, and then ST239 separately.

Excluding ST239, the CC8 sample consists of 18 MRSA isolates (red branches), ten MSSA isolates (green branches) and five reference genomes. The CC8 tree reveals two related,

but distinct, star-like radiations encompassing 17 MRSA SCC*mec* type IV

isolates and two MSSA. This pattern indicates a single acquisition of type IVc SCC*mec* in the common ancestor of these two clusters, followed by the loss of this element in the two MSSA isolates in one of the clusters and the diversification into the closely related subtypes IVa and IVx in the other cluster. Both clusters are geographically restricted to Belgium and France, with the exception of three isolates within the smaller cluster from Italy, Croatia and Spain. A single MRSA isolate from Belgium clusters closely with the two USA300 reference genomes. The presence of the type I arginine catabolic mobile genetic element (ACME) encoding the arginine deaminase pathway, and the φSa2usaprophage encoding Panton-Valentine leucocidin (PVL) (see also supplementary Figure 6) strongly suggests that this isolate represents a USA300 isolate.
