# Supplementary material for: Whole-Genome Sequencing for Routine Pathogen Surveillance in Public Health: a Population Snapshot of Invasive Staphylococcus aureus in Europe
Source: mBio. 2016 May 5;7(3):e00444-16. doi: 10.1128/mBio.00444-16 (PMC4959656; doi:10.1128/mBio.00444-16)
Supplement: Figure S3 — (A) Phylogenetic reconstruction of ST239 isolates in the sample. Red branches depict all of the MRSA isolates. The symbols at the tips of the branches indicate the geographic origins of the isolates. Isolates carrying the sasX gene are indicated by the letter X. Blue shading highlights isolates belonging to the Asian clade according to Harris et al. (S. R. Harris, et al., Science 327:469-474, 2010). (B) Phylogenetic tree of combined ST239 data obtained from isolates from the reports of Harris et al. and Castillo-Ramírez et al. (S. Castillo-Ramírez, et al., Genome Biol 13:R126, 2012) and isolates in panel A. Clusters are named in accordance with the reports of Harris et al. and Castillo-Ramírez et al. Download [file mbo002162813sf3.pdf]

ST239

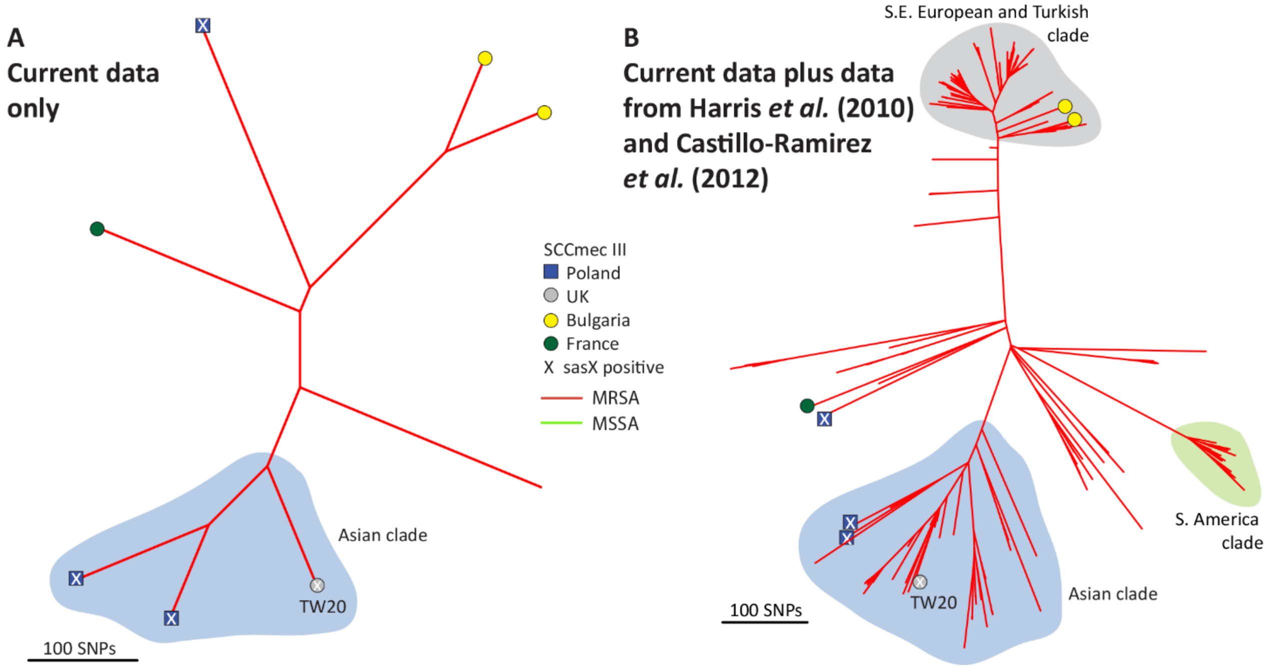

All of the six ST239 isolates in the sample (Figure 7A) have acquired the large SCC*mec* type III element that is characteristic of this multi-resistant, healthcare-associated clone. Two of the Polish ST239 isolates are closely related to the TW20 reference [22] that corresponds to the 'Asian clade' of ST239 [54], indicating an Oriental origin of these Polish isolates. Members of the Asian clade harbour the φSPβ-like prophage that encodes SasX, a cell wall- anchored surface protein, which is linked to the epidemiological success of ST239 in China and S.E. Asia [41]. This phage, and the *sasX* gene, are also found in the two Polish isolates which cluster with TW20, again supporting the Asian origin of these isolates (Figure S1). Combining the ST239 data with previously published ST239 data [54,56] confirmed the position of these two Polish isolates within the Asian clade (Figure 7B). This analysis also revealed that the third Polish isolate, and the single ST239 isolate from France, are phylogenetically distinct. Surprisingly, this third Polish isolate also contains a φSPβ-like prophage encoding *sasX* despite not being part of the Asian clade. This phage was likely to have been acquired from an Asian-derived ST239 population co-circulating in Poland. To our knowledge this is the first example of the horizontal dissemination, presumably via lysogenic conversion, of the phage-borne *sasX* virulence factor within Europe.
