# Supplementary material for: Whole-Genome Sequencing for Routine Pathogen Surveillance in Public Health: a Population Snapshot of Invasive Staphylococcus aureus in Europe
Source: mBio. 2016 May 5;7(3):e00444-16. doi: 10.1128/mBio.00444-16 (PMC4959656; doi:10.1128/mBio.00444-16)
Supplement: Figure S4 — Phylogenetic reconstruction of CC15. Starlike phylogeny indicates the recent rapid expansion of this MSSA-only lineage. Symbols at the tips indicate the geographic origins of these isolates. The branch leading to an ST582 isolate—a single-locus variant of CC15—is shortened, and the number of SNPs defining that branch is shown. Download [file mbo002162813sf4.pdf]

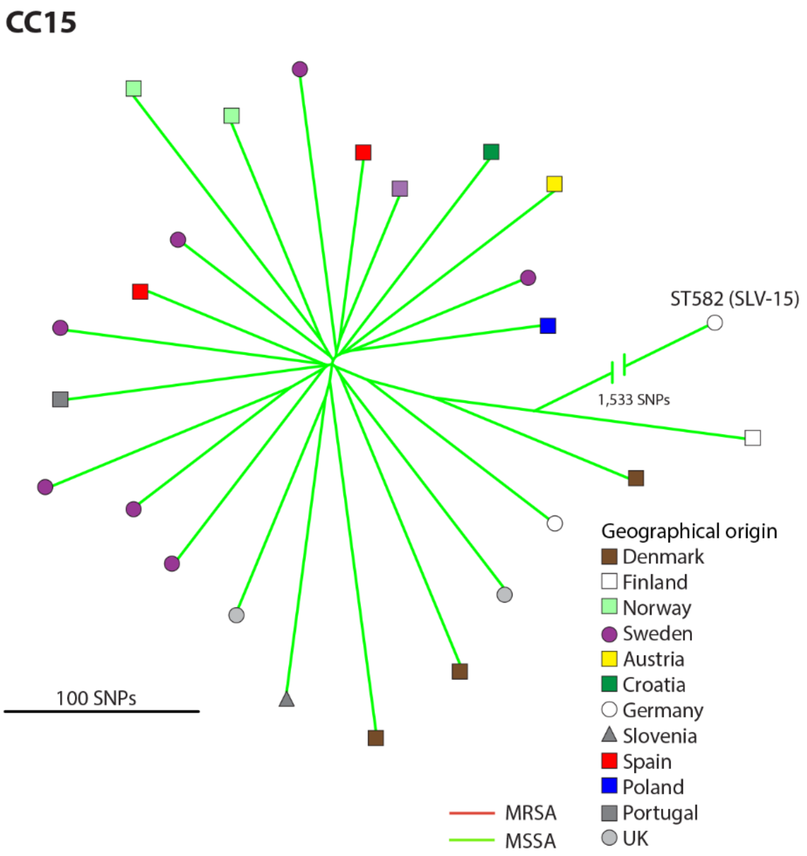

CC 15 consists of 25 MSSA isolates (green branches), no MRSA and no reference genomes. Excluding a single highly diverged genome corresponding to ST582, it is the least diverse CC (Table 1). The tree reveals a star-like phylogeny indicative of rapid recent expansion from a common ancestor, but very little geographic clustering. The diverged isolate, ST582, from Germany falls outside the main CC15 radiation. This isolate is a single locus variant (SLV)* of ST15, and closer inspection of the genome data revealed that it has acquired a large chromosomal replacement affecting approximately 10% of the genome (See below). A striking feature of CC15 is the paucity of MGEs and accessory genes, as discussed in the main text.

Large Homologous Recombination

We noted a very large homologous recombination event within strain 296_DE_582 which is positioned just outside the main CC15 cluster and originates from Germany. This isolate corresponds to ST582, which is an SLV of ST15, differing from this genotype by 4 SNPs within the *tpi* gene. There is a single example of this ST582 in the MLST database, corresponding to a strain

recovered in Portugal in 2003. The transferred SNPs extend from base 778798 to 1081494 (positions relative to EMRSA15 reference), meaning that the minimum size is 302696-Kb bp (~10% of the genome). The recombined region encompasses the *tpi* MLST gene, and also the pathogenicity island SAPI1. There are 1328 SNP differences between 296_DE_582 and the CC15 isolate 297_FI_2874 (ST2874) in this 302-Kb region, corresponding to 0.44% divergence. We identified isolate 291_SE_20 as the isolate in our dataset as the most closely related to the original donor. 291_SE_20 is an ST20 isolate that differs at only 26 SNP sites (0.0085%) from 296_DE_582 within the imported block, a 50 fold lower level of divergence than that observed between 296_DE_582 and other CC15 isolates within this genomic region. This region thus represents an almost base- perfect "cut-and-paste" recombination event resulting in the acquisition of over 302-Kb from ST20 into an ST15 background. This novel large replacement has affected a different region of the genome from those previously described in *S. aureus*, such as the one in ST239, which is situated closer to the origin of replication. The figure shows two sliding window diversity plots between the hybrid genome (ST582) vs the minor parent (ST20 donor) and the major parent (ST15 recipient).
