# Supplementary figures and images for: Whole-Genome Sequencing for Routine Pathogen Surveillance in Public Health: a Population Snapshot of Invasive Staphylococcus aureus in Europe
Source: mBio. 2016 May 5;7(3):e00444-16. doi: 10.1128/mBio.00444-16 (PMC4959656; doi:10.1128/mBio.00444-16)

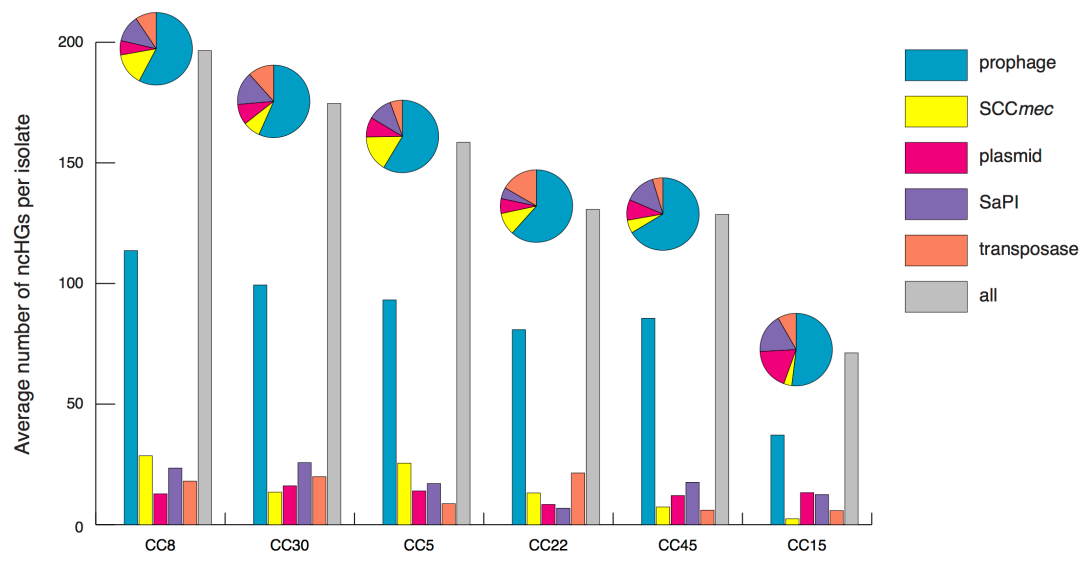

Supplement: Figure S5 — Number of ncHGs per major lineage. The bar chart shows the average number of ncHGs per isolate split into MGE type for each of the six major lineages. Each pie chart indicates the proportion of MGE types for each major lineage. Download [file mbo002162813sf5.pdf]

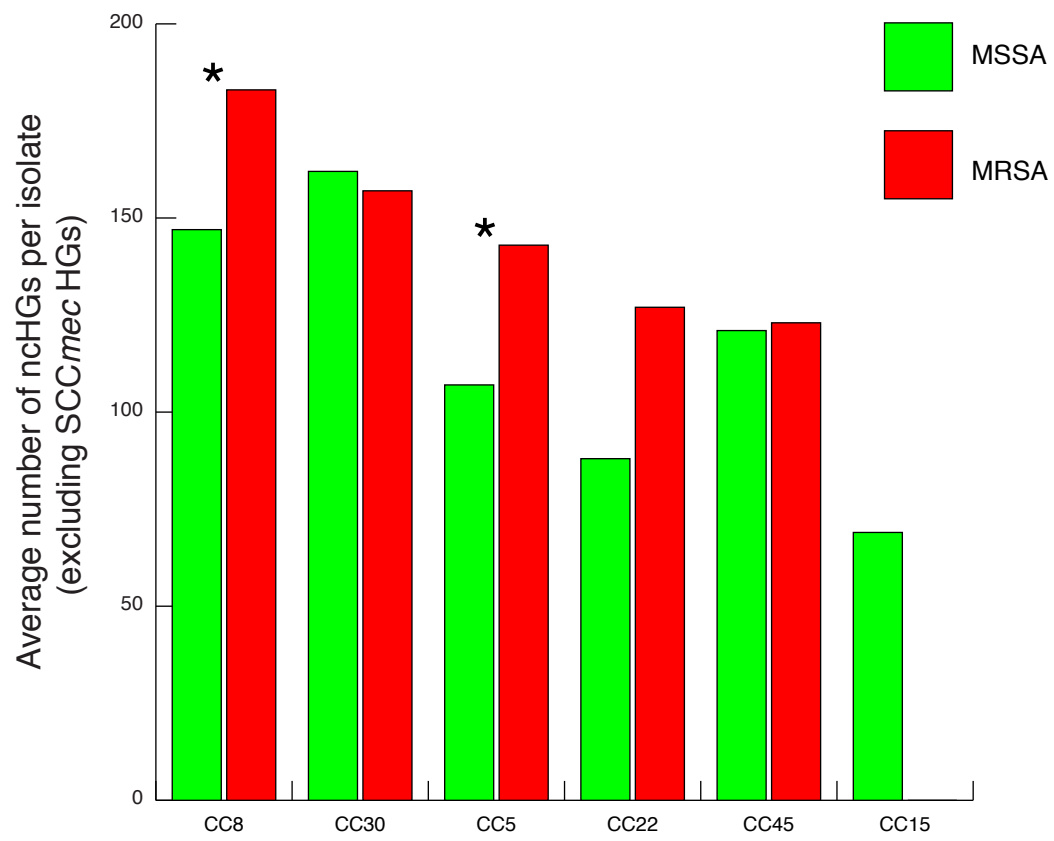

Supplement: Figure S6 — MRSA or MSSA genome size. The bar chart shows the average number of ncHGs per MSSA or MRSA isolate, respectively, excluding the HGs associated with SCCmec, for each major lineage. Asterisks indicate lineages with a significant size difference of the accessory genome in MSSA and MRSA isolates. CC8, P = 0.039; CC5, P = 0.0325 (statistically significant); CC22, P = 0.0539 (not quite statistically significant). Download [file mbo002162813sf6.pdf]

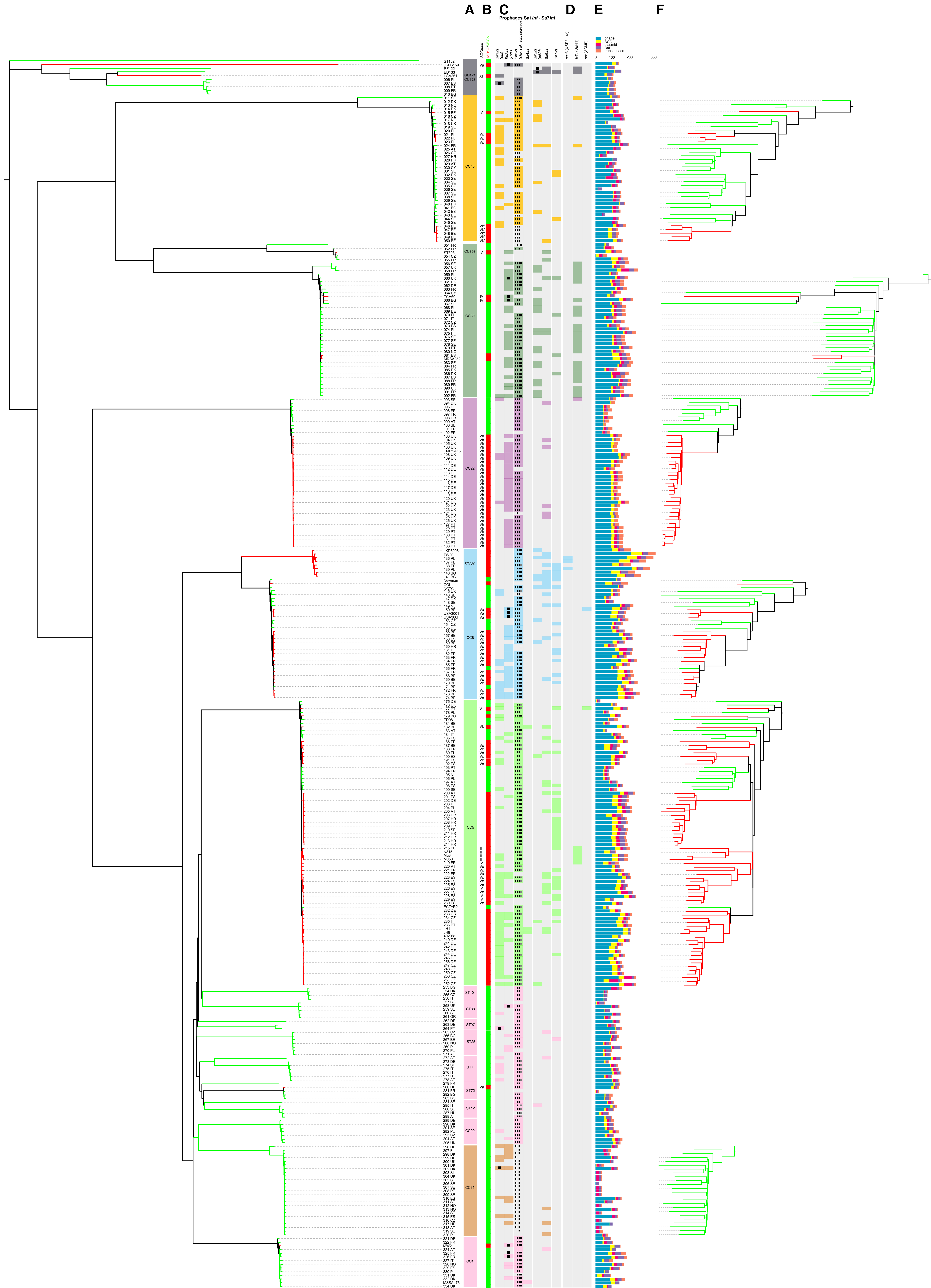

Supplement: Figure S7 — Prophage distribution. Rooted neighbor-joining tree like that in Fig. 1. Colors of branches indicate MSSA (green) and MRSA (red). Each isolate is annotated for affiliation to CCs or STs (A), SSCmec type and MSSA (green) or MRSA (red) (B), and seven prophage types classified on the basis of the presence or absence of their integrase genes (C). Colored boxes indicate presence. Black boxes indicate the presence of virulence genes associated with the prophage. (D) Colored boxes indicate the presence of two virulence genes (sasX and tstH) and acr—the gene commonly used to detect ACMEs. (E) Size and composition of the accessory genome based on the number of ncHGs with further categorization according to MGE type. (F) Close-up of phylogenetic trees of the six major lineages. Download [file mbo002162813sf7.pdf]
